# Lipid Surfaces and Glutamate Anions Enhance Formation of Dynamic Biomolecular Condensates Containing Bacterial Cell Division Protein FtsZ and Its DNA-Bound Regulator SlmA

**DOI:** 10.1021/acs.biochem.2c00424

**Published:** 2022-10-31

**Authors:** Gianfranco Paccione, Miguel Á. Robles-Ramos, Carlos Alfonso, Marta Sobrinos-Sanguino, William Margolin, Silvia Zorrilla, Begoña Monterroso, Germán Rivas

**Affiliations:** † Centro de Investigaciones Biológicas Margarita Salas, Consejo Superior de Investigaciones Científicas (CSIC), 28040 Madrid, Spain; ‡ Department of Microbiology and Molecular Genetics, McGovern Medical School, University of Texas, Houston, Texas 77030, United States

## Abstract

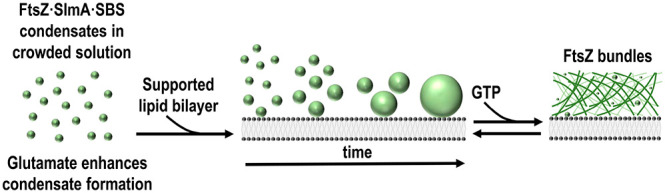

Dynamic biomolecular condensates formed by liquid–liquid phase separation can regulate the spatial and temporal organization of proteins, thus modulating their functional activity in cells. Previous studies showed that the cell division protein FtsZ from *Escherichia coli* formed dynamic phase-separated condensates with nucleoprotein complexes containing the FtsZ spatial regulator SlmA under crowding conditions, with potential implications for condensate-mediated spatiotemporal control of FtsZ activity in cell division. In the present study, we assessed formation of these condensates in the presence of lipid surfaces and glutamate ions to better approximate the *E. coli* intracellular environment. We found that potassium glutamate substantially promoted the formation of FtsZ-containing condensates when compared to potassium chloride in crowded solutions. These condensates accumulated on supported lipid bilayers and eventually fused, resulting in a time-dependent increase in the droplet size. Moreover, the accumulated condensates were dynamic, capturing protein from the external phase. FtsZ partitioned into the condensates at the lipid surface only in its guanosine diphosphate (GDP) form, regardless of whether it came from FtsZ polymer disassembly upon guanosine triphosphate (GTP) exhaustion. These results provide insights into the behavior of these GTP-responsive condensates in minimal membrane systems, which suggest how these membraneless assemblies may tune critical bacterial division events during the cell cycle.

Research over the past decade has revealed the existence of numerous membrane-less intracellular assemblies (often termed biomolecular condensates), in many cases tentatively identified as immiscible liquid phases likely driven by phase transitions analogous to liquid–liquid phase separations.^1^ These dynamic structures can concentrate one or more proteins, nucleic acids, and other biomolecules away from the surrounding milieu, providing unique microenvironments to modulate or control the rates and equilibria of critical biochemical reactions and assembly processes. Proteins that contain multivalent domains, such as those involved in protein–protein and protein–nucleic acid interactions, and those containing intrinsically disordered regions are prone to form these condensates.^2^ Excluded volume effects related to natural crowding can promote these phase-separation processes.^3−5^

Most biomolecular condensates that have been characterized are from eukaryotic systems, with fundamental roles in cellular organization, physiology (e.g., metabolic regulation, signaling, gene expression, stress adaptation, etc.), and pathology (e.g., aberrant condensates linked to age-related diseases).^6^ However, recent progress has revealed that condensates are also widespread in prokaryotic systems and are associated with essential processes such as chromosome segregation, DNA compaction/repair, mRNA transcription/degradation, etc.^7−10^

The FtsZ protein, a central element of the division machinery (namely, the divisome) in most bacteria, is also susceptible to reversible partitioning into dynamic condensates. In *Escherichia coli*, FtsZ interacts with additional proteins to form a ring at midcell that is essential to constrict the cytoplasmic membrane inward for cytokinesis.^11^ FtsZ does not bind to the cytoplasmic membrane on its own and therefore needs other proteins for attachment. The FtsZ ring assembles by GTP-dependent polymerization of FtsZ into protofilaments, which in *E. coli* are tethered to the membrane by FtsA and ZipA proteins. FtsZ protofilaments lose subunits preferentially at one end upon GTP hydrolysis, resulting in processive circumferential migration of protofilaments around the ring by treadmilling.^12^ Several site-selection mechanisms precisely control the location of the FtsZ ring to midcell during the cell cycle to safeguard genome integrity and bacterial survival.^13^ Two of these, the Min system and the nucleoid occlusion system mediated by the SlmA protein, prevent inappropriate assembly of FtsZ rings near the cell poles and over the nucleoid, respectively. SlmA is a DNA-binding protein that specifically binds to several chromosomal DNA sequences called SlmA-binding sites (SBSs). SBS-bound SlmA is an inhibitor of FtsZ polymerization and thus prevents FtsZ ring formation in the vicinity of the nucleoid.^14^

FtsZ assembles into dynamic phase-separated condensates in vitro under crowding conditions resembling the volume occupancy of the cell interior, both in bulk or encapsulated in cell-like lipid containers.^15^ FtsZ condensate formation is strongly favored in the presence of SBS-bound SlmA.^16^ Interestingly, the GDP-bound FtsZ species are preferentially partitioned into the condensates. These condensates subsequently disband upon the addition of GTP, driving the formation of FtsZ protofilaments and filament bundles. GTPase-driven conversion of bound GTP into GDP disassembles FtsZ polymers, causing reassembly of the FtsZ-containing condensates.

The formation of these FtsZ·SlmA·SBS condensates may have potential physiological implications. Before cell division, SlmA·SBS may recruit FtsZ and form the condensates, enhancing the localization of these membrane-less assemblies near the cytoplasmic membrane. Once the cell is ready for division, FtsZ will be recruited to midcell to polymerize into a “Z-ring” driven by GTP.

The aforementioned studies of phase-separated FtsZ condensates in vitro have been performed in potassium chloride buffers; in contrast, glutamate is the most relevant and abundant physiological anion in *Escherichia coli*.^17^ Although it has been shown that FtsZ maintains its self-association properties in potassium glutamate,^18^ replacing chloride with glutamate significantly affects bacterial protein–DNA associations.^19^ Moreover, recent studies report that glutamate promotes liquid–liquid phase separation and DNA-binding cooperativity of *E. coli* SSB protein.^20^

Previous results have shown that FtsZ·SlmA·SBS condensates accumulate at the lipid interface when encapsulated in lipid-stabilized microdroplets.^16^ Interestingly, SlmA binds to lipid membranes, as recently revealed by biochemical membrane-reconstitution approaches.^21^ These findings suggest that membrane surfaces may control biomolecular phase separation in bacteria, as shown already in several eukaryotic systems,^22^ and may help to modulate FtsZ spatiotemporal organization inside bacterial cells.

In this work, we have investigated the effects of these two physiologically relevant factors—glutamate and lipid surfaces—on the behavior of droplet-like condensates formed by FtsZ when mixed with SlmA and DNA oligonucleotides harboring SBS. The assays were done in the presence of high concentrations of Ficoll 70 to reproduce the crowded cell interior. We found that glutamate and lipid surfaces enhance FtsZ·SlmA·SBS condensate formation in a time-dependent manner at the lipid interface.

## Materials and Methods

### Materials

GTP, salts, and buffer components of analytical grade were from Sigma. Ficoll 70 (GE Healthcare) was dialyzed prior to its use in 20 mM Tris-HCl, pH 7.5, and the final concentration was determined from its refractive index increment (0.141 mL/g).^23^*Escherichia coli* polar extract phospholipids (Avanti Polar Lipids) were kept in spectroscopic-grade chloroform (Merck) at −20 °C. Fluorescently labeled DOPE (ATTO655-DOPE) was from ATTO-Tech GmbH. Specific single-stranded SBS oligonucleotides, unlabeled or labeled with Alexa Fluor 647 at the 5′ end when required, were from Integrated DNA Technologies. Single-stranded oligonucleotides containing a single consensus sequence (5′-AA**GTAAGTGAGCGCTCACTTAC**GT-3′, bases recognized by SlmA in bold)^14^ were hybridized as previously described.^24^

### Protein Purification and Labeling

FtsZ and SlmA were purified as earlier stated.^14,25^ When required, FtsZ was covalently labeled with Alexa Fluor 488 carboxylic acid succinimidyl ester dye (Invitrogen) as previously described.^18,26^ Labeling degree, estimated through spectroscopic quantification, ranged between 0.3 and 0.9 mol of fluorophore per mole of protein. Proteins were stored at −80 °C until used.

### Turbidity Measurements

Turbidity measurements were conducted as in ref (15). Briefly, signal was measured at 350 nm in a Varioskan Flash plate reader (Thermo) at room temperature in 96-well half area plates (Corning). Samples contained 12 μM FtsZ, 5 μM SlmA, and 1 μM SBS in KCl (50 mM Tris-HCl pH 7.5, 150, 300, or 500 mM KCl, and 5 mM MgCl_2_) or KGlu (20 mM Tris-HCl pH 7.5, 150, 300, 400, 500, or 600 mM KGlu, and 5 mM MgCl_2_) with 100 g/L Ficoll 70 in a final volume of 100 μL. All samples were incubated for 30 min prior to their measurement. The results are the average of four independent measurements ± standard deviation (SD).

### Supported Lipid Bilayer Formation

Supported lipid bilayers (SLBs) were prepared following the protocol described in ref (27) with minor modifications. Briefly, the *E. coli* polar lipid extract in chloroform was dried in a Speed-Vac desiccator for 1 h. The dry lipid film was rehydrated in 50 mM Tris-HCl pH 7.5 and 150 mM KCl (SLB buffer) to a final 4 g/L concentration and sonicated to obtain small unilamellar vesicles. A plastic chamber was glued to a glass coverslip (previously cleaned by sonication in 80% ethanol) by applying an ultraviolet-curable glue (Norland optical adhesive, Norland Products) to the bottom of the chamber and subsequently was exposed to an ultraviolet light source for 15 min. The vesicle suspension was diluted to a final 0.5 g/L concentration in the SLB buffer, and 75 μL were loaded in each chamber. To promote vesicle fusion and lipid bilayer formation, 2 mM CaCl_2_ was added to each chamber. After a 20 min incubation at 37 °C, the excess of lipid and calcium was removed by washing the lipid bilayer with 2 mL of prewarmed SLB buffer. Fluorescently labeled SLBs were formed by adding 0.05% mol of ATTO655-DOPE to the mixture of *E. coli* lipids in chloroform.

### Confocal Microscopy Sample Preparation and Data Analysis

Images were obtained with a LEICA TCS SP5 AOBS inverted confocal microscope equipped with a HCX PL APO 63x oil immersion objective (na 1.4, Leica). The excitation lines were 488 (Ar laser) for FtsZ-Alexa 488 and 633 (He–Ne laser) for SBS-Alexa 647 and ATTO655-DOPE. Transmission light (DIC) and fluorescence images were taken simultaneously. Upon SLB formation, buffer exchange was done by washing the lipid container with the final buffer composition, including crowder (Ficoll 70, 300 g/L stock solution). Samples with SBS (50 μM stock solution), SlmA (150 μM stock solution), and/or FtsZ (250 μM stock solution), containing 0.5 μM FtsZ-Alexa 488 and/or 0.5 μM SBS-Alexa 647, were directly added on top of the SLB in that order. Finally, the sample (100 μL final volume) was homogenized by mixing the upper half of the sample volume. All samples were incubated for 30 min prior to their visualization unless otherwise stated. For polymerization experiments, samples were prepared with all components including 1 mM GTP, except for FtsZ, which was added just before imaging. Protein-capture experiments to assess protein exchange between condensates and the outer phase were conducted by adding FtsZ-Alexa 488 to preformed condensates labeled with SBS-Alexa 647 after a 30 min incubation and then slowly stirring the sample for 5 min to allow the protein to diffuse to the lipid bilayer without resuspending the condensates on the lipid surface.

ImageJ^28^ was used (1) to evaluate droplet fusion through time-lapse analysis, (2) to acquire the intensity profiles relative to the distance from the lipid bilayer, (3) to obtain orthogonal views (YZ) of the lipid bilayer from a Z-stack of images, and (4) to measure the average particle sizes over time. Particle analysis employed the ImageJ particle analysis tool excluding particles on the edges and with a circularity <0.7 after reducing noise by applying a Kuwahara filter and threshold correction.

## Results

**Figure 1:**
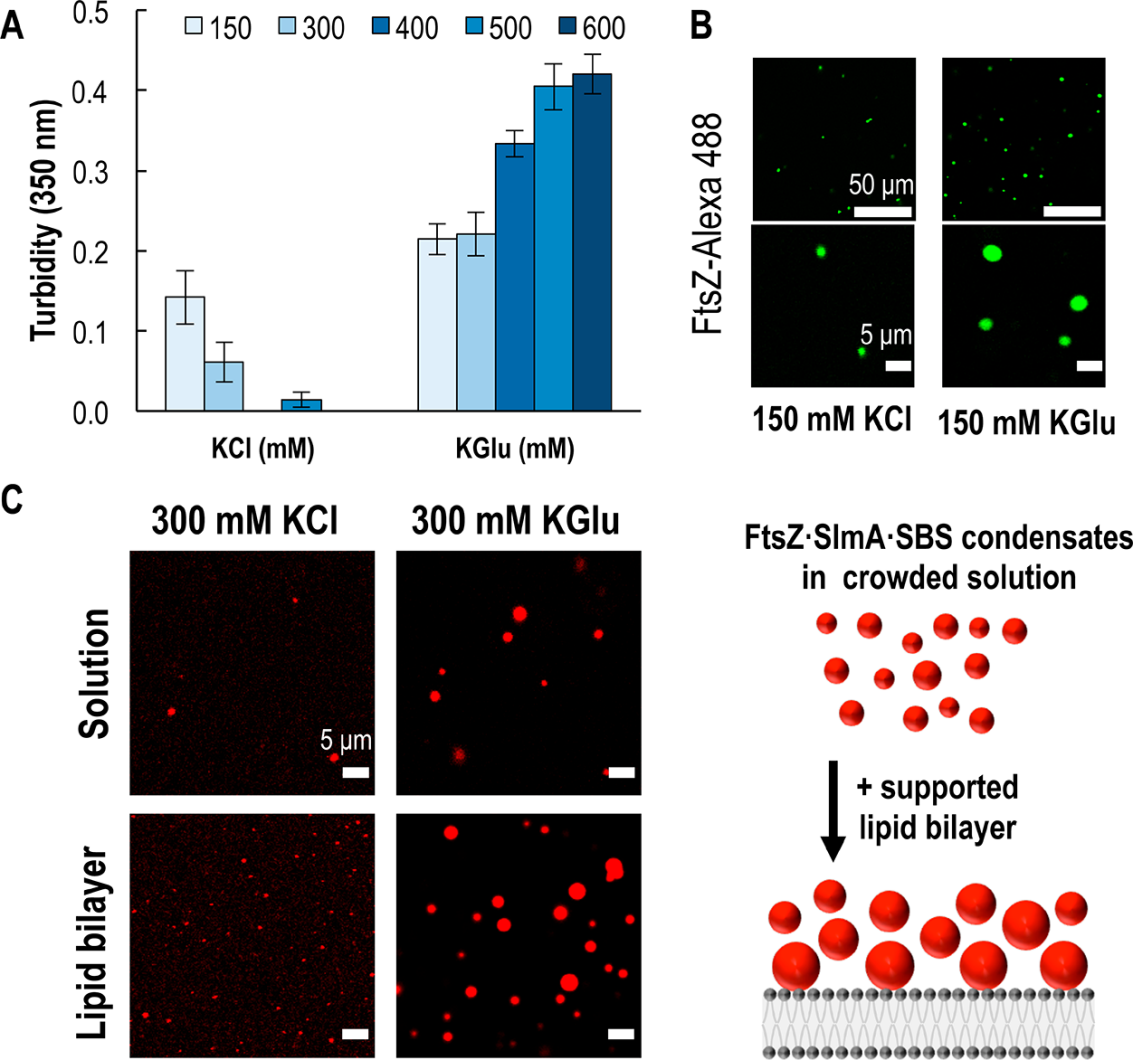
Formation of FtsZ·SlmA·SBS condensates in potassium glutamate (KGlu) and potassium chloride (KCl). (A) Turbidity signal of FtsZ·SlmA·SBS in varying salt conditions. Data correspond to the average of four independent experiments ± SD. (B) Representative confocal images of FtsZ·SlmA·SBS condensates (FtsZ-Alexa 488) in solution in 150 mM KCl or KGlu at two different magnifications. (C) Representative confocal images (left) of FtsZ·SlmA·SBS condensates (SBS-Alexa 647) in 300 mM KCl or KGlu in the presence or absence of a supported lipid bilayer, and scheme illustrating the accumulation of condensates on the membrane (right). All experiments with 12 μM FtsZ, 5 μM SlmA, and 1 μM SBS with 100 g/L Ficoll 70, 5 mM Mg^2+^, and pH 7.5.

In an attempt to use more physiological buffer conditions for condensate formation, we compared the effects of potassium glutamate (KGlu) on the formation of FtsZ·SlmA·SBS condensates with those of KCl. These conditions containing KGlu resemble the ionic composition of the *E. coli* cytoplasm more faithfully. We used turbidity assays to monitor the formation of droplet-like condensates in crowded conditions (100 g/L Ficoll 70) containing FtsZ and SlmA·SBS, as increased formation of these structures correlates with higher turbidity.^16^ We observed significant increases in the turbidity signal in KGlu compared to KCl at all salt concentrations tested (Figure 1A), which is compatible with higher abundance and/or larger size of the condensates. The increase in the turbidity as glutamate concentration increases indicates that glutamate anions enhance condensate formation. In contrast, as chloride concentration increases, condensate formation decreases, in agreement with previous data implicating electrostatic interactions involved in forming condensates.^16^

Furthermore, confocal images showed an increase in the number and size of the droplets formed in KGlu compared to equivalent samples in KCl (Figure 1B and C). Together, these data suggest that KGlu enhances the formation of phase-separated condensates with FtsZ, SlmA, and SBS in crowded conditions.

**Figure 2:**
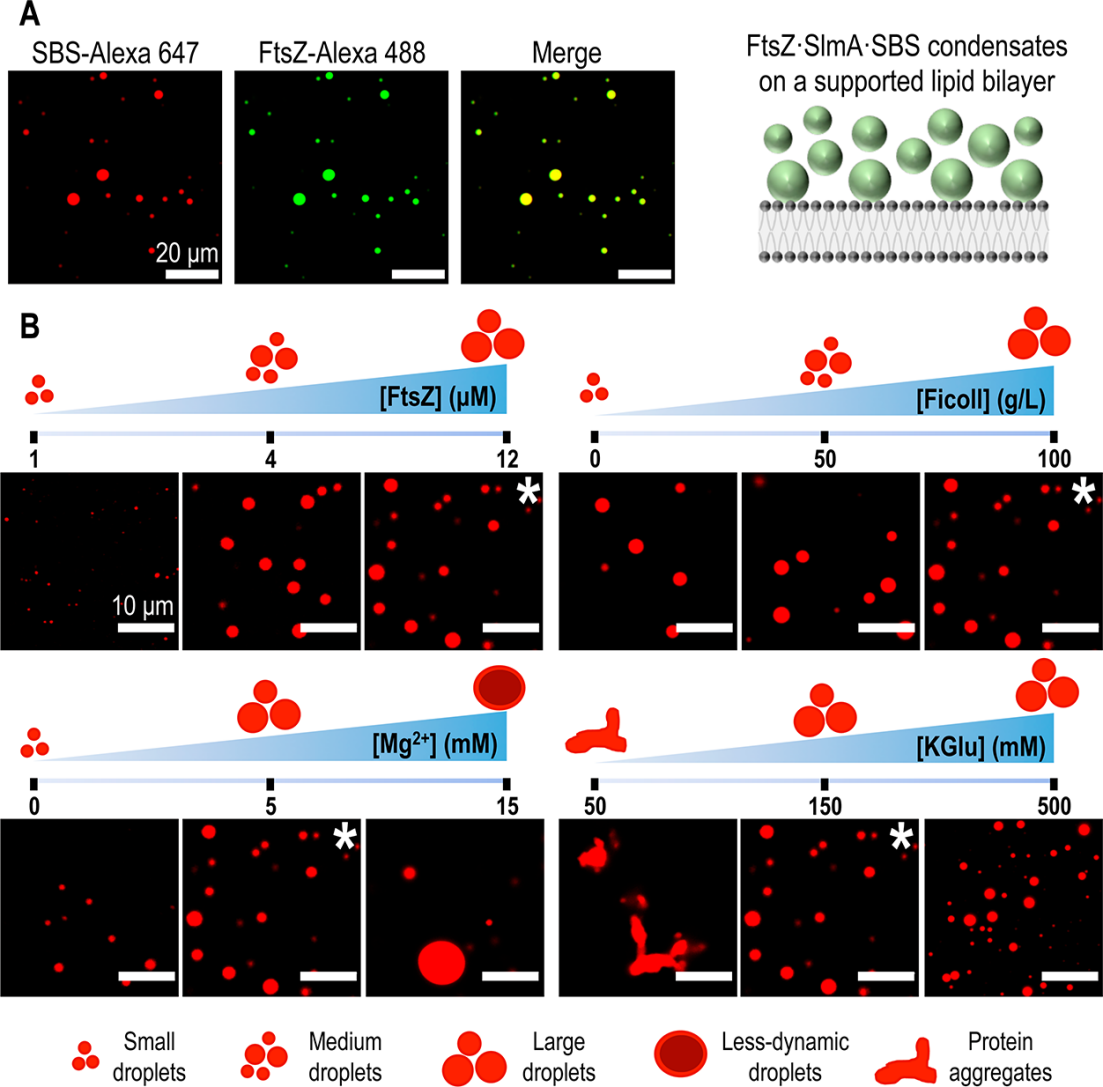
Formation of FtsZ·SlmA·SBS condensates on a supported lipid bilayer. (A) Colocalization of SlmA·SBS and FtsZ in the condensates in standard conditions and scheme illustrating the formation of condensates on the membrane on the right. (B) Effect of varying conditions in FtsZ·SlmA·SBS condensates (with SBS-Alexa 647) formed on a supported lipid bilayer. Images marked with an asterisk correspond to the standard condition (12:5:1 μM FtsZ:SlmA:SBS and 100 g/L Ficoll 70 with 5 mM Mg^2+^ in 150 mM KGlu buffer, pH 7.5). For each parameter, schemes illustrating the effects observed are included.

Next, we characterized the effect that a lipid surface might have on the formation and properties of the condensates. To answer this question, we initially tested the influence of preparing samples at equal KCl or KGlu concentrations (300 mM) in the presence or absence of a supported lipid bilayer. We observed a synergic effect of glutamate and the lipid bilayer, resulting in an accumulation of the condensates at the lipid interface (Figure 1C). To assess the effect of the different variables affecting condensate formation, we chose an initial condition (12:5:1 μM FtsZ/SlmA/SBS and 100 g/L Ficoll 70 with 5 mM Mg^2+^ in 150 mM KGlu buffer, pH 7.5), henceforth used as the standard condition, and then varied each parameter individually in screening assays that compared confocal microscopy images of samples on supported lipid bilayers (Figure 2). As with higher KGlu content, we observed condensate formation at the lipid bilayer under this salt condition, where fluorescently tagged FtsZ and SBS (FtsZ-Alexa 488 and SBS-Alexa 647) colocalized (Figure 2A). FtsZ and SlmA·SBS did not form condensates on their own (Figure S1), in agreement with previous assays in the absence of lipid surfaces.^16^ Lipid bilayers containing a small amount of fluorescently labeled lipid tracer allowed for an orthogonal view of the droplet accumulation at the membrane (Figure S2). We observed that the droplets accumulated in the space between the membrane surface and ∼10 μm above it.

Decreasing crowder (Ficoll 70), protein (FtsZ), and Mg^2+^ concentrations resulted in a decreased formation and accumulation of the FtsZ-containing droplets on the lipid interface according to a visual analysis of the images (Figure 2B). Although the magnitude of the effect differed depending on the parameter varied, they all showed a similar trend. Notably, unlike what was previously observed in solution,^16^ condensates were also formed in the absence of crowder (Figure 2B), highlighting the enhancing effect of KGlu and the lipid surface on their assembly. Increasing the Mg^2+^ concentration resulted in an increase in condensate size with a subsequent decrease in abundance. The effect of glutamate was less obvious here than that described in the turbidity assays in solution (Figure 1A). Between 150 and 500 mM KGlu, there is a slight increase in condensate abundance on the lipid surface as glutamate concentration increases, without an apparent impact on their size, compatible with the idea that glutamate enhances condensate formation. Protein aggregation observed at low ionic strengths (50 mM KGlu) is consistent with previous findings in which macromolecular condensates are obtained at intermediate ionic strengths, but, upon reaching a certain threshold, lower ionic strengths result in irregular protein aggregate formation.^29^

**Figure 3:**
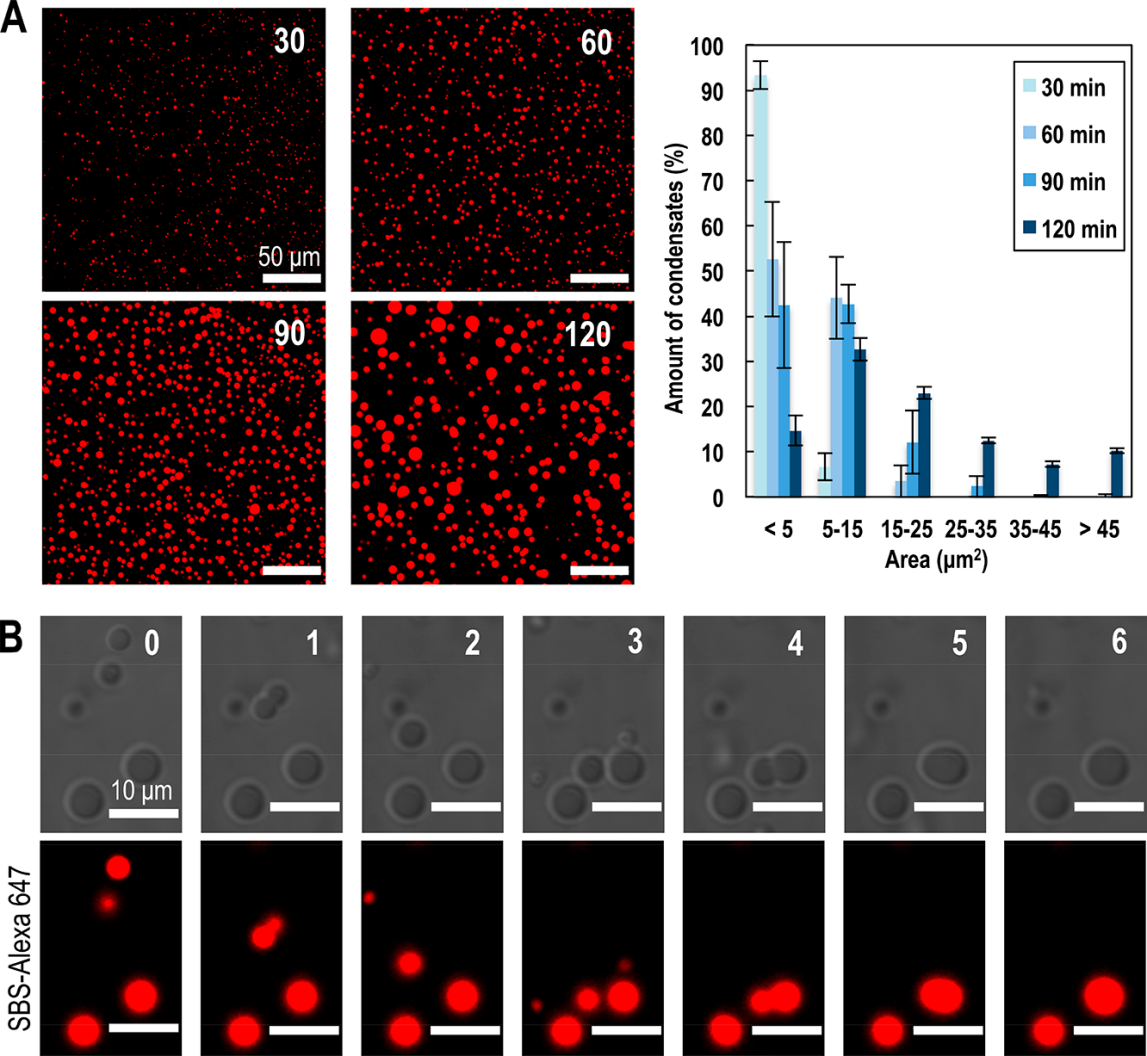
Time evolution and fusion of FtsZ·SlmA·SBS condensates on a supported lipid bilayer. (A) Representative fluorescence confocal images of the FtsZ·SlmA·SBS condensates (SBS-Alexa 647) on the bilayer at the indicated incubation times in min (left) and quantification of the size distribution (*n* = 1076, 1169, 1562, and 571 for 30, 60, 90, and 120 min, respectively) from the images (right). Errors correspond to the SD from 2 independent experiments. (B) Stepwise fusion of several FtsZ·SlmA·SBS (SBS-Alexa 647) condensates for a total time lapse of 6 min. All experiments were performed in standard conditions.

We then investigated the kinetics of condensate formation in greater detail, at the initial standard conditions previously mentioned. We monitored the accumulation of droplet-like condensates containing FtsZ and SlmA·SBS over time in the vicinity of the lipid bilayer, as revealed by confocal microscopy images taken at 30 min intervals for 2 h (Figure 3A). Over time, the quantitative analysis of these images showed a shift in the size distribution of the condensates accumulated on the lipid interface. After 30 min incubation, the average diameter of the whole population is 1.6 ± 0.7 μm, with more than 90% condensates having an area <5 μm^2^. Likewise, at an elapsed time of 120 min, the average diameter of the condensates is 5.2 ± 2.4 μm, with <20% of the condensates having an area <5 μm^2^ (Figure 3A). Interestingly, these droplets were capable of fusing to form larger ones as observed in the 6 min time lapse presented in Figure 3B. In general, droplets interacting with the lipid bilayer were in a relatively fixed position. This facilitated the monitoring of fusion events occurring between two condensates accumulated on the lipid surface and those between a droplet in the solution settling on a droplet on the lipid surface, increasing the average size of particles accumulated on the lipid surface over time.

Earlier work showed that the phase-separated condensates formed by FtsZ and SlmA·SBS in solution are dynamic, as they could exchange externally added protein and, more interestingly, reversibly evolve into FtsZ fibers in the presence of GTP.^16^ Here, the accumulation of the droplet-like condensates on the lipid surface made it more challenging to experimentally test the dynamic properties of the condensates, requiring further optimization.

**Figure 4:**
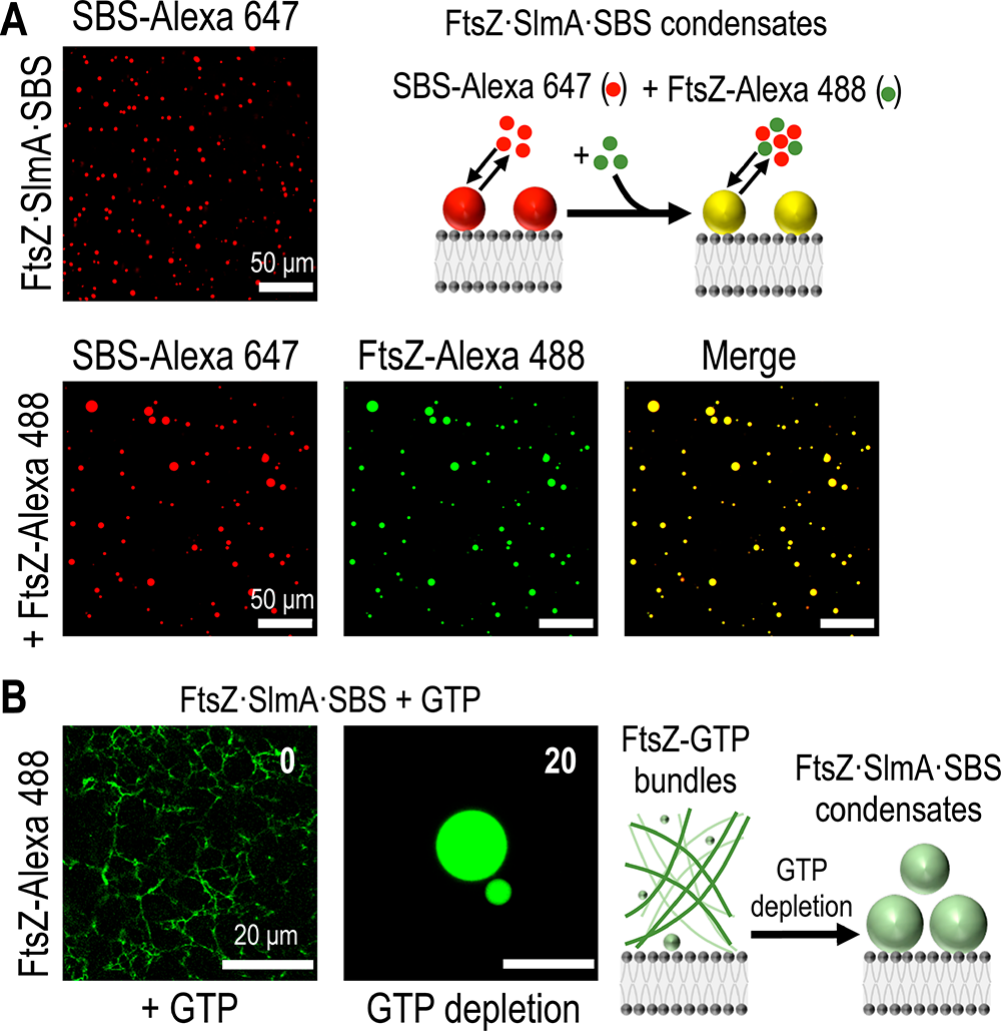
Dynamism of FtsZ·SlmA·SBS condensates on a supported lipid bilayer. (A) Representative confocal images showing the initial (top) and final (bottom) state after addition of FtsZ-Alexa 488 to preformed condensates with SBS-Alexa 647. The scheme depicts the incorporation into the condensate of externally added FtsZ-Alexa 488. (B) Polymers formed by FtsZ (FtsZ-Alexa 488) in the presence of SBS-bound SlmA and 1 mM GTP, and reassembly of condensates after GTP depletion. Time from GTP addition in min is indicated. An illustration of the process is on the right. All experiments were performed in standard conditions.

First, to show that these droplets were dynamic and could recruit new protein added to the mixture, thorough but careful homogenization of the protein mixture was required so that the newly added protein would reach the lipid bilayer without altering the accumulated droplets on the surface. We used slow stirring to let the protein diffuse to the lipid bilayer without resuspending the condensates on the lipid surface. This procedure allowed us to acquire images demonstrating that droplets formed in standard conditions had incorporated the externally added protein (Figure 4A). Parallel experiments performed with the droplets formed at a high Mg^2+^ concentration showed a reduced ability to capture newly added protein, having a lower protein exchange at higher Mg^2+^ concentrations, suggesting that they are less dynamic (Figure S3). Consistent with this idea, we also observed slower dynamics in higher Mg^2+^ concentrations in fluorescence recovery after photobleaching (FRAP) experiments (Figure S4).

We then tested if GTP controls the reversible transition between FtsZ fibers and condensates through the association state of FtsZ, determined by the bound nucleotide (GDP or GTP). Indeed, addition of GTP under standard conditions triggered the formation of FtsZ fibers that disassembled upon GTP depletion due to FtsZ GTPase activity. Subsequently, the FtsZ-GDP oligomers condensed into droplets together with SlmA and the SBS oligonucleotide, as visualized by confocal microscopy in samples containing fluorescently labeled FtsZ (Figure 4B). A time-evolution experiment of the disassembly of FtsZ polymers and formation of FtsZ·SlmA·SBS condensates is shown Figure S5. Together with the fusion events shown earlier, these results indicate that the condensates formed by FtsZ and SlmA·SBS at the surface of supported bilayers are dynamic, a characteristic of liquid-like droplets formed by phase separation.

## Discussion

Understanding the phase-separation behavior of FtsZ in the context of physiologically relevant elements of the bacterial cell, such as KGlu and lipid surfaces under crowded conditions, should help elucidate the nature of these GTP-responsive condensates and their potential role in regulating bacterial cell division. This work has provided evidence that glutamate enhances the assembly of FtsZ and SlmA·SBS complexes into condensates through associative liquid–liquid phase separation (LLPS) under crowding conditions. The use of supported lipid bilayers as minimal membrane systems has demonstrated that these condensates accumulate in the vicinity of the membrane; they are dynamic, and they reversibly evolve into FtsZ fibers in the presence of GTP.

These observations may shed light on the mechanism by which the active form of SlmA (bound to SBS) antagonizes Z-ring assembly. Because condensates are dynamic, FtsZ in the condensate (and presumably the other components in the condensate with their counterparts in solution) exchanges with FtsZ in the bulk phase. Adding GTP to the solution phase stimulates fiber formation by FtsZ both from the pool (which favors FtsZ in the condensates returning to the solution phase) and from the condensate, leading to gradual fiber formation in the solution phase and depletion of the condensate. When the GTP is exhausted, the FtsZ fibers disassemble and the released FtsZ pool recondenses with SlmA and SBS. Which state predominates under a particular set of conditions will depend on the balance of factors favoring condensation (i.e., crowding and membrane surfaces) and fiber formation (i.e., GTP concentration).

Several lines of evidence suggest that this equilibrium between FtsZ·SlmA·SBS condensates and FtsZ polymers in solution has a role in *E. coli* cell division. First, as glutamate is a more physiological anion than chloride inside the *E. coli* cytoplasm, the enhanced ability of FtsZ and SlmA·SBS to form condensates in the presence of KGlu compared with KCl points to physiological relevance of the condensates. Second, GTP levels can drastically decrease under stress conditions, connected to the activation of (p)ppGpp signaling, for example, upon antibiotic treatment. These observations suggest that FtsZ-containing condensates might play a significant role in bacterial resistance to antibiotics. In addition to genetically acquired antibiotic resistance, bacteria can survive antibiotic stress through the existence of a subset of cells that become tolerant to antibiotics, denoted as persister cells, whose mechanisms of formation are poorly understood.^30^ The reversible displacement of FtsZ from the condensed phase to form fibers driven by GTP would explain in part how persister cells can resume growth and division once stress conditions are alleviated and GTP concentration increases to physiological levels.

In support of this idea, during nutrient starvation FtsZ relocalizes into focal aggregates at *E. coli* cell poles that may be condensates.^31^ These foci disassemble and convert to Z-rings once growth resumes, presumably in concert with a rise in GTP levels in response to increased nutrient levels, which may reflect the reversible release of FtsZ from condensates into polymers that we observe in vitro. It is noteworthy that condensates of SpmX-PopZ proteins in *Caulobacter crescentus* bacteria regulate the localization of a key protein kinase involved in cell division (DivJ) that binds to ATP, and these SpmX-PopZ condensates can be specifically dissolved by the addition of ATP.^32^ This ATP-dependent dissolution of protein condensates affects the localization of DivJ and thereby regulates cell cycle signaling in response to nutrient availability. We propose that FtsZ·SlmA·SBS is another example of dissolution of a bacterial protein condensate in response to nutrient levels, only in this case GTP levels are used instead of ATP. There are also several eukaryotic examples of ATP-dependent dissolution of protein condensates, which seem to require the negatively charged triphosphate moiety of ATP in the process.^33,34^ Therefore, it seems reasonable that a similar mechanism may occur with GTP levels regulating formation of condensates containing a GTP binding protein such as FtsZ.

Our studies have shown that Mg^2+^ controls some of the properties of the FtsZ condensates, as increasing the concentrations of this divalent cation led to increasing size and decreasing abundance and dynamic behavior of the condensates. A possible explanation for this behavior might be an increase in the system viscosity at higher Mg^2+^ concentrations, in line with previous findings that link Mg^2+^ concentrations with changes in the fluidity of condensates.^35^ Further analysis will be required to determine the viscoelastic properties of these condensates and their dependence on physiologically relevant ligands, such as Mg^2+^ and nucleotides.

Our experimental approach employs a supported lipid bilayer to study condensate–membrane interactions, which is relevant in the field as recent work shows.^36,37^ This approach can easily be extended to incorporate other divisome proteins to investigate their effects on the formation of FtsZ-containing biomolecular condensates. Moreover, further studies are underway to understand the role of the other positive and negative regulators of Z-ring stability on the assembly and properties of FtsZ-containing condensates. Such future work will contribute to a better understanding of the role of phase separation in the temporal and spatial regulation of Z-ring assembly in bacterial cells, particularly under different environmental conditions.

## Supporting Information Available

Individual behavior of SlmA·SBS and FtsZ on a supported lipid bilayer; orthogonal view of the FtsZ·SlmA·SBS condensates; Z-plot profile of mean fluorescence intensities; protein-capture experiment of condensates in 15 mM Mg^2+^; FRAP experiments of condensates in standard conditions and 15 mM Mg^2+^; and time evolution of FtsZ polymers in the presence of SlmA·SBS and GTP (PDF)

## Accession Codes

FtsZ (*E. coli*): P0A9A6; SlmA (*E. coli*): P0C093
