# Supplementary material for: Lipid Surfaces and Glutamate Anions Enhance Formation of Dynamic Biomolecular Condensates Containing Bacterial Cell Division Protein FtsZ and Its DNA-Bound Regulator SlmA
Source: Biochemistry. 2022 Oct 31;61(22):2482–9. doi: 10.1021/acs.biochem.2c00424 (PMC9670838; doi:10.1021/acs.biochem.2c00424)
Supplement: Supplementary file 1 — bi2c00424_si_001.pdf [file bi2c00424_si_001.pdf]

# Supporting Information

## Lipid surfaces and glutamate anions enhance formation of dynamic biomolecular condensates containing bacterial cell division protein FtsZ and its DNA-bound regulator SlmA

*Gianfranco Paccione*[§], *Miguel Á. Robles-Ramos*[§], *Carlos Alfonso*[§], *Marta Sobrinos-Sanguino*[§], *William Margolin*[¶], *Silvia Zorrilla*[§‡]*, *Begoña Monterroso*[§‡]*, *Germán Rivas*[§]*

[§] Centro de Investigaciones Biológicas Margarita Salas, Consejo Superior de Investigaciones Científicas (CSIC), 28040-Madrid, Spain

[¶] Department of Microbiology and Molecular Genetics, McGovern Medical School, University of Texas, Houston, TX 77030, USA

LIST OF FIGURES

Figure S1 …………………………………………………………………………………...... S1

Figure S2 …………………………………………………………………………………...... S2

Figure S3 …………………………………………………………………………………...... S3

Figure S4 …………………………………………………………………………………...... S4

Figure S5 …………………………………………………………………………………...... S5

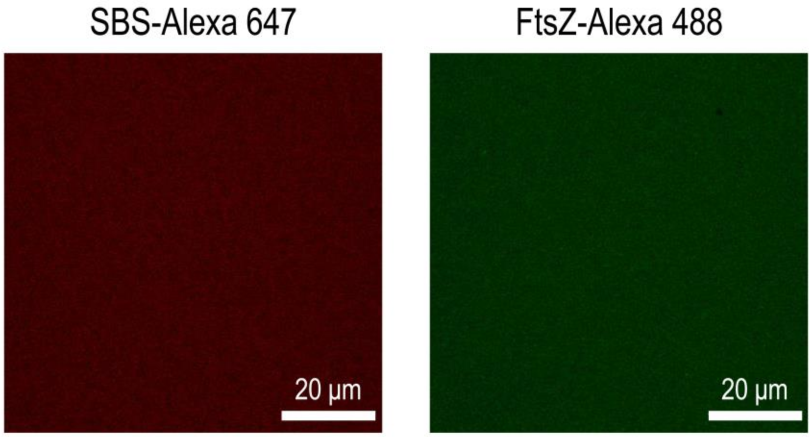


**Figure S1.** Individual behavior of SlmA·SBS (in the absence of FtsZ, left) and FtsZ (in the absence of SlmA·SBS, right) on a supported lipid bilayer. Fluorescence confocal microscopy images of samples in standard conditions, with the indicated elements.

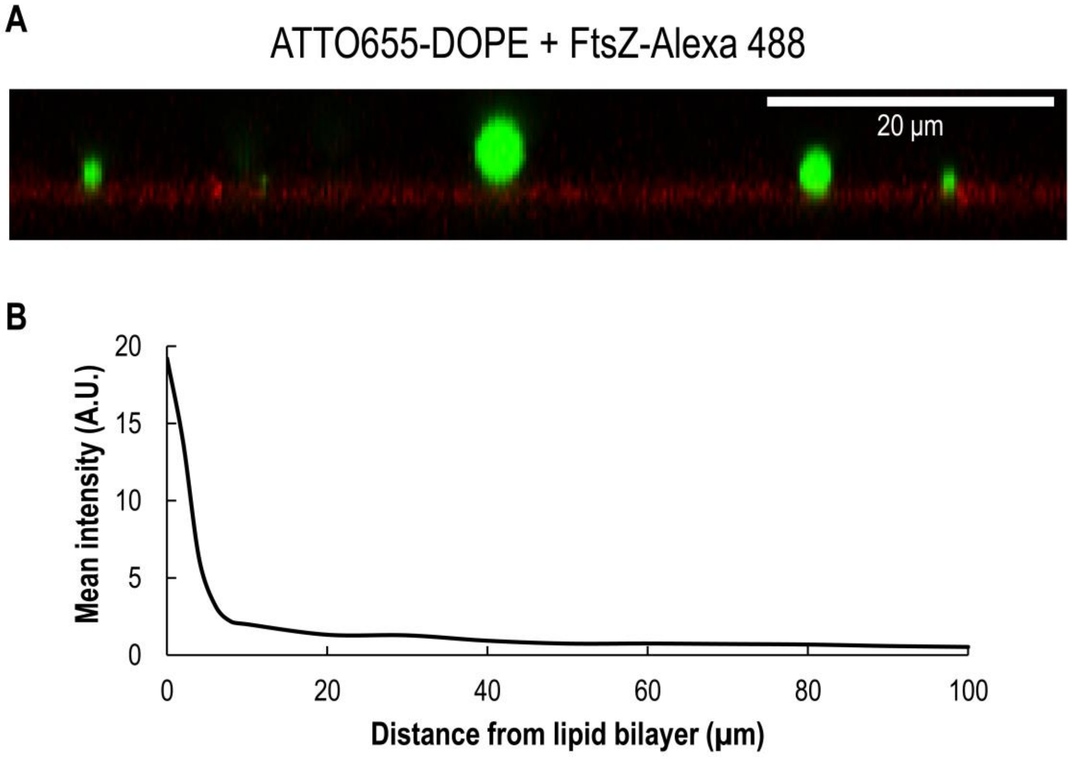


**Figure S2**. Arrangement of the FtsZ·SlmA·SBS condensates on a supported lipid bilayer. (A) Orthogonal view (YZ) of a Z-stack of fluorescence confocal microscopy images. Condensates in standard conditions. Lipid bilayer labeled with 0.05% mol ATTO655-DOPE. (B) Z-plot profile of the mean intensity of fluorescence images of FtsZ·SlmA·SBS droplets in standard conditions, after 60-min incubation.

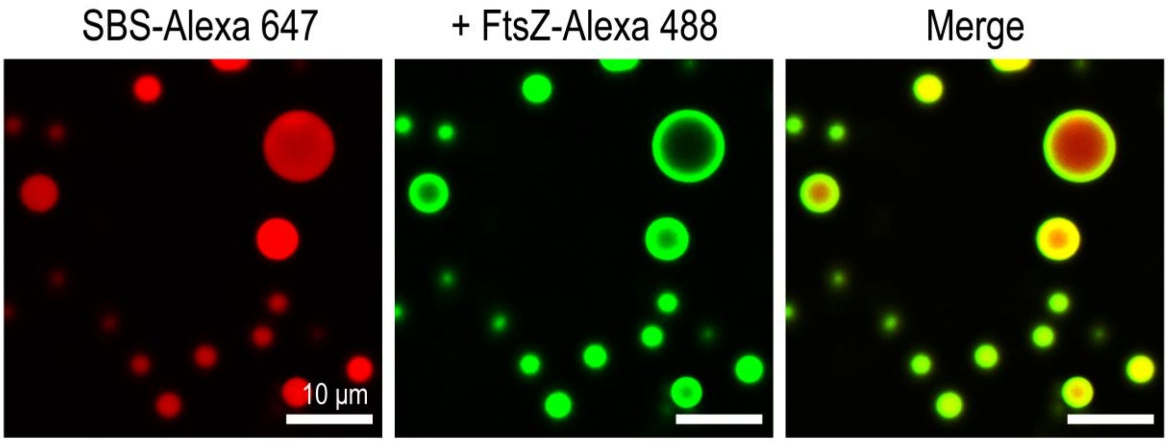


**Figure S3**. Reduced dynamics of condensates formed in 15 mM $Mg^{2+}$ on a supported lipid bilayer. Fluorescence confocal microscopy images show the addition of FtsZ-Alexa 488 to FtsZ·SlmA·SBS condensates labeled with SBS-Alexa 647 formed in standard conditions but with 15 mM $Mg^{2+}$ instead of 5 mM $Mg^{2+}$.

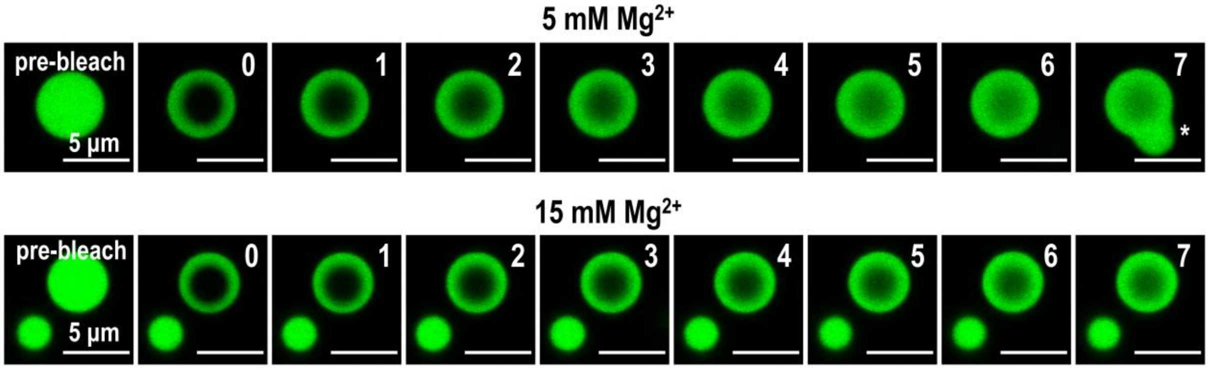


**Figure S4**. FRAP experiments to monitor the dynamics of the condensates under different conditions. Confocal microscopy images of similarly-sized condensates in standard conditions (top row), and with 15 mM $Mg^{2+}$ (bottom row). Samples contained 0.5 µM FtsZ-Alexa 488. Pre-bleach and recovery images are included. Time in minutes after bleaching is indicated on each image. Bleaching is performed at 60% laser power with 16 iterations, whereas pre-bleach and recovery images are obtained at 10% laser power. Recovery images are obtained every 60 s. Indicated with an asterisk, a fusion event can be observed after 7 minutes in standard conditions (5 mM $Mg^{2+}$), after which images are not included due to fusion events obscuring fluorescence recovery evaluation.

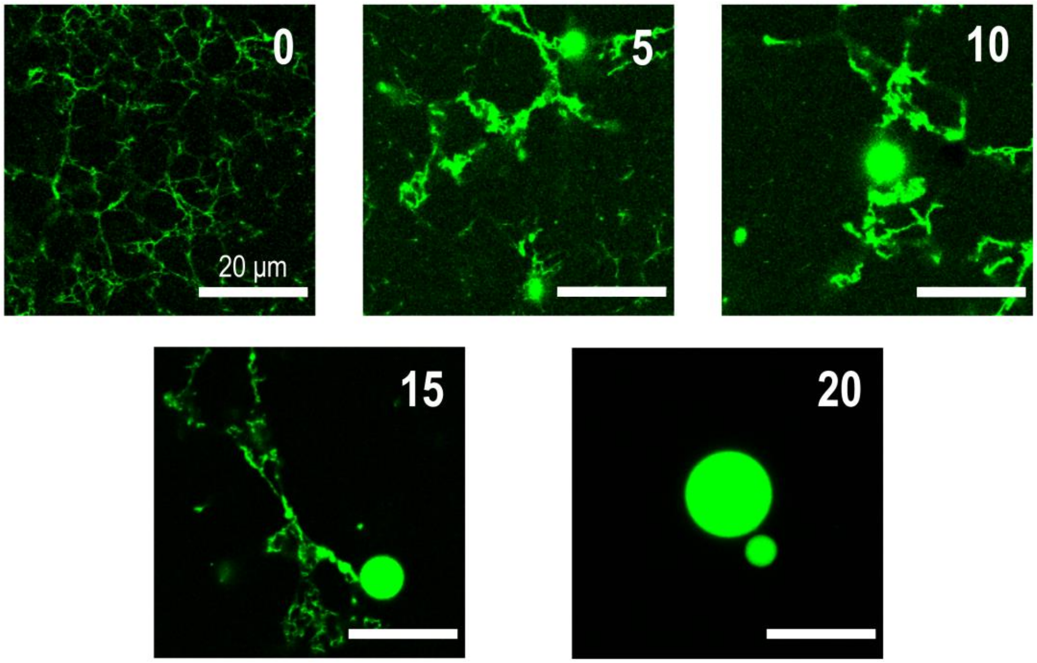


**Figure S5**. Time-evolution of polymers formed by FtsZ (FtsZ-Alexa 488) in the presence of SBS-bound SlmA and 1 mM GTP on a supported lipid bilayer in standard conditions. Time from FtsZ addition in minutes is indicated. The top row images were acquired with a higher gain to better visualize the polymers, while the bottom row show images acquired with lower gain to better visualize the condensates.
